# Evidence on User-Led Innovation in Diabetes Technology (The OPEN Project): Protocol for a Mixed Methods Study

**DOI:** 10.2196/15368

**Published:** 2019-11-19

**Authors:** Shane O'Donnell, Dana Lewis, María Marchante Fernández, Mandy Wäldchen, Bryan Cleal, Timothy Skinner, Klemens Raile, Adrian Tappe, Tebbe Ubben, Ingrid Willaing, Bastian Hauck, Saskia Wolf, Katarina Braune

**Affiliations:** 1 School of Sociology University College Dublin Belfield Ireland; 2 OpenAPS Seattle, WA United States; 3 Diabetes Management Research Steno Diabetes Center Copenhagen Gentofte Denmark; 4 Institut for Psykologi Københavns Universitet Copenhagen Denmark; 5 Department of Paediatric Endocrinology and Diabetes Charité - Universitätsmedizin Berlin Berlin Germany; 6 AndroidAPS Vienna Austria; 7 #dedoc° Diabetes Online Community Berlin Germany

**Keywords:** diabetes, digital health, open source, closed-loop insulin delivery systems, automated insulin delivery systems, #WeAreNotWaiting

## Abstract

**Background:**

Digital innovations in health care have traditionally followed a top-down pathway, with manufacturers leading the design and production of technology-enabled solutions and those living with chronic conditions involved only as passive recipients of the end product. However, user-driven open-source initiatives in health care are becoming increasingly popular. An example is the growing movement of people with diabetes, who create their own “Do-It-Yourself Artificial Pancreas Systems” (DIYAPS).

**Objective:**

The overall aim of this study is to establish the empirical evidence base for the clinical effectiveness and quality-of-life benefits of DIYAPS and identify the challenges and possible solutions to enable their wider diffusion.

**Methods:**

A research program comprising 5 work packages will examine the outcomes and potential for scaling up DIYAPS solutions. Quantitative and qualitative methodologies will be used to examine clinical and self-reported outcome measures of DIYAPS users. The majority of members of the research team live with type 1 diabetes and are active DIYAPS users, making *Outcomes of Patients’ Evidence With Novel, Do-It-Yourself Artificial Pancreas Technology* (OPEN) a unique, user-driven research project.

**Results:**

This project has received funding from the European Commission’s Horizon 2020 Research and Innovation Program, under the Marie Skłodowska-Curie Action Research and Innovation Staff Exchange. Researchers with both academic and nonacademic backgrounds have been recruited to formulate research questions, drive the research process, and disseminate ongoing findings back to the DIYAPS community and other stakeholders.

**Conclusions:**

The OPEN project is unique in that it is a truly patient- and user-led research project, which brings together an international, interdisciplinary, and intersectoral research group, comprising health care professionals, technical developers, biomedical and social scientists, the majority of whom are also living with diabetes. Thus, it directly addresses the core research and user needs of the DIYAPS movement. As a new model of cooperation, it will highlight how researchers in academia, industry, and the patient community can create patient-centric innovation and reduce disease burden together.

**International Registered Report Identifier (IRRID):**

PRR1-10.2196/15368

## Introduction

Type 1 diabetes (T1D) is a challenging chronic condition, which often leads to lowered life expectancy and diminished quality of life [1]. Despite significant advances in insulin therapy and technological developments, only 17% of youth and 21% of adults with diabetes achieve a glycated hemoglobin (HbA_1c_) level of <7.0% (58 mmol/mol), as recommended in clinical guidelines [2,3].

### Closing the Loop: Automated Insulin Delivery Systems

In general, closed-loop insulin delivery systems, also called “automated insulin delivery systems (AID)” or “artificial pancreas systems (APS),” combine sensors for continuous glucose monitoring (CGM) and insulin pumps with a control algorithm, and these are characterized by automated insulin delivery in response to the user’s glucose level. As subcutaneously administered insulin stays active for multiple hours, the algorithm used to calculate the amount of insulin needed has to predict future glucose values to operate safely. Closed-loop systems designed for commercial use have been shown to be safe and effective in reducing hyperglycemia and hypoglycemia in people with diabetes (PwD) of all age groups [4-8], and these systems are therefore seen as the gold standard of future diabetes therapy [9].

Qualitative research on commercially developed closed-loop systems has indicated that individuals using these systems for relatively short periods report reduced anxiety [10-14], improved quality of sleep [10,14-17], and reduced burden of managing diabetes [12,13,17-19] and this led to greater freedom and flexibility in their lifestyle as a result [12,16-18]. This is supported by a few quantitative studies that report less fear of hypoglycemia [10-13,20,21]—although possibly because of small sample sizes, the changes are not consistently significant—a reduction in diabetes-specific distress in 2 studies [13,20], and, in a single study, improved sleep quality [22]. These studies have used a range of closed-loop or technology-specific quality-of-life instruments, with the Diabetes Technology Questionnaire being the most widely used.

### The Do-It-Yourself Artificial Pancreas: #WeAreNotWaiting

However, although a variety of commercial APS are under development and some have recently become available in a limited number of countries, they are not universally available, accessible, or affordable. Behind the hashtag #WeAreNotWaiting, a community of PwD and their families have created new tools and systems to help PwD better utilize their devices and data. These systems are co-created in the Do-It-Yourself Artificial Pancreas System (DIYAPS) community, but each user has to build their own individual system themselves and use at their own risk. Instructions and code for these systems have been made universally available via open-source platforms [23]. The DIYAPS or “Open-Source Artificial Pancreas System” (OpenAPS) is one of the most significant developments to emerge through this movement [24]. In these systems, insulin delivery is automated and remotely controlled by open-source algorithms and by reverse engineering and connecting commercially available and approved insulin pumps and CGM systems. The term “open source” describes software whose source code is publicly available. Open-source licenses usually deny liability and warranty and may require disclosing source code and referring to the project [25].

Initial observational studies on DIYAPS have described significant improvements in glycemic control, quality of life, and sleep quality in DIYAPS users of all age groups, including children and adolescents, where caregivers build and maintain these systems on their behalf [26-31]. A limited number of studies are also specifically reporting on the experience of using DIYAPS, and in addition to highlighting improved sleep [28,30,32] and reduced burden of diabetes management [32], they point to increased confidence, increased energy, and reduced mood swings [32].

Evidence of usage of DIYAPS is limited to date, as none of these systems have yet been evaluated by a randomized controlled trial, regarding safety and efficiency—although at least 1 is planned [33]. Observational studies largely describe outcomes self-reported by users and are mainly based on smaller cohort studies (up to n=80) [27]. There is an estimated 15+ million hours of real-world DIYAPS data, much of which have yet to be fully analyzed. A global investigation based on DIYAPS data is of interest to inform potential users of these systems regarding the benefits and challenges of using the system and to learn more about how clinical and quality-of-life outcomes are affected by different groups of DIYAPS users, as well as the mechanisms through which these results are being achieved.

Another fundamental question is who might get left behind in this user-driven technological innovation. DIYAPS aims to better target the complexity of diabetes self-management for the person with diabetes, reduce the cognitive and emotional burden on PwD, and improve clinical outcomes. Such an outcome would make an important contribution to reducing inequalities in outcomes that are linked to individual disparities in the capacity to cope with these burdens. However, there are numerous challenges to be overcome to achieve this objective, and the complexity of establishing and maintaining effective DIYAPS currently remains high for many PwD. Thus, a further challenge with respect to social inequality rests in how to ensure that the benefits of APS are widely diffused across the population so that no one is left behind in their diabetes care. Therefore, the challenges in this area are not exclusively medical or technical, but also ethical, sociological, and political in nature and require an interdisciplinary and intersectoral approach to be addressed effectively. Moreover, there is a rich vein of expertise and knowledge available from nontraditional experts within the DIYAPS community, which has traditionally been overlooked by both academia and industry. Successfully bringing this nontraditional expertise into mainstream health care settings is key to addressing some of the core research opportunities and challenges that are likely to emerge as do-it-yourself (DIY) solutions become increasingly popular and shape digital innovations in diabetes care.

### Objectives of the OPEN Project

Thus, the aim of the OPEN project (*Outcomes of Patients’ Evidence With Novel, Do-It-Yourself Artificial Pancreas Technology*) is to examine what academia, industry, and PwD can learn from one another, with the goal of making artificial pancreas technology of all kinds available to everyone. The OPEN consortium achieves this by bringing together an intersectoral and interdisciplinary research team comprising patient innovators, academic researchers in biomedical and social sciences, health care professionals, and patient advocacy organizations to establish an empirical evidence base surrounding the impact of DIYAPS.

This collaboration is facilitated through a series of staff exchanges between high-profile nonacademic organizations dedicated to patient-driven approaches (Steno Diabetes Center Copenhagen, Denmark; Dedoc Labs, Germany) and leading research organizations in the field of diabetes research and connected health (Charité—Universitätsmedizin Berlin, Germany; University College Dublin, Ireland).

The central aims and objectives of the OPEN project have been developed on the basis of the priorities of the DIYAPS and wider diabetes communities. Furthermore, a key goal of the OPEN project is to tap into the expertise of the DIYAPS community to bring their knowledge and expertise to mainstream health care settings.

OPEN is already co-led by some of the key members of the DIYAPS movement and will actively continue to involve members of the DIYAPS and wider diabetes communities to facilitate their participation in OPEN, via staff secondments and further collaborations. Furthermore, members of the Diabetes Online Community are being recruited to assist with disseminating the findings of the project on an ongoing basis and maintain a consistent dialogue between OPEN and the wider diabetes community.

## Methods

### Overview

A total of 5 interdependent work packages (WPs) have been proposed: The first 2 WPs are focused on acquiring data to demonstrate what, if any, are the clinical, quality-of-life, and psychosocial benefits of DIYAPS. This will include engaging the community in sharing glucose and insulin dosing data, as well as self-reporting on their experience of living with DIYAPS. WP3 is focused on reducing the technical barriers to DIYAPS. This also feeds into the work of WP4, which is identifying the barriers to wider uptake of these user-led innovations and exploring ways to reduce them. The final WP relates to how the OPEN project will disseminate the results to the research, health care, and community of PwD.

### Work Package 1: Clinical Outcomes and Guidelines

The purpose of this WP is to evaluate the clinical outcomes of DIYAPS users of all age groups globally and create a draft for future guidelines for closed-loop technology in clinical routine. Individuals using DIYAPS (any type) have the ability to anonymously donate their data to research projects, via the OpenAPS Data Commons, on the citizen science platform “Open Humans” [31]. Users specifically consent to share their data for research purposes, and they can choose to either manually upload data of their choice or upload data via an upload tool of their choice, with the data source of choice (for further details, please see the description of WP3). The OPEN team will request access to and utilize data from the OpenAPS Data Commons, which is considered pseudonymized for purposes of the OPEN project’s use.

Glycemic outcomes will be analyzed in a pre-post evaluation of prospectively collected data from DIYAPS users. CGM sensor data will be analyzed to calculate certain parameters, such as the Time in Range (percentage of sensor glucose levels between 70 mg/dL and 180 mg/dL) before and after DIYAPS initiation as a primary key endpoint, as well as Time below Range (<70 mg/dL, <54 mg/dL) and Time above Range (>180 mg/dL, >250 mg/dL) as secondary endpoints. Other secondary key endpoints include self-reported parameters, such as HbA_1c_ levels, incidence of acute diabetes-related complications, such as severe hypoglycemic events and incidence and possible cause of diabetic ketoacidosis. Assessment of basic demographic and health data, such as specifications of diabetes treatment, socioeconomic status, gender, age, weight, height, comorbidities, and incidence of diabetes-related complications, will enable analysis of clinical outcomes for different user groups. To further validate accuracy of self-reported clinical data from patients and caregivers, data from a subcohort will be clinically compared with independent medical data repositories to add to the evidence base regarding the reliability of real-world data.

This WP is led by KB and KR, both PwDs and medical doctors at the Department of Pediatric Endocrinology and Diabetes at Charité University Medicine Berlin.

### Work Package 2: Patient-Reported Outcomes

Alongside work to evaluate the clinical benefits of DIYAPS, we will seek to establish the quality of life and lived experiences of DIYAPS users. Given the lack of an internally consistent, reliable, sensitive, and validated T1D-specific quality-of-life questionnaire that also uses item wording that is widely acceptable to people with T1D [34], the project will take a facet approach to assessing the quality-of-life outcomes. For DIYAPS users and their primary caregivers and loved ones, we will assess the potential benefits of DIYAPS on emotional well-being, sleep quality, hypoglycemia-related anxiety and fears, the burden of diabetes, and flexibility of lifestyle. Furthermore, we will investigate motivations, barriers, and retention factors to building and maintaining DIYAPS in a questionnaire-based survey and whether there are additional benefits accrued by individuals assembling their own closed-loop systems [35,36]. We will also explore effects on individuals’ sense of self-efficacy, social support, and the benefits accrued from joining a wider network of people with T1D.

In addition to the survey data on quality-of-life outcomes, the project will also undertake qualitative research with DIYAPS users to generate data about their lived experience with this technology. An objective here is to examine how the lived experiences of DIYAPS users vary across socioeconomic status, gender, ethnicity, and age. To achieve this, a purposively sampled select group of the project’s participants will be asked to describe the day-to-day burden associated with T1D and DIYAPS. Such burdens might include but not be limited to out-of-pocket expenses, ability to carry out daily tasks in the work setting or at home, participation in social activities and other issues related to social connectedness, informal care provided by family members or relatives, and episodes of distress caused by living with diabetes. 

This WP is led by IW, BC, and TS, diabetes management researchers at Steno Diabetes Center Copenhagen.

### Work Package 3: Technical Development

This WP will focus on the evaluation and possible improvements of DIYAPS through statistical and machine learning techniques. The WP has 2 main objectives: (1) improving ease of use for DIYAPS users’ data donation to support further research and evaluation and (2) evaluating existing DIYAPS platforms and implications for APS improvement.

We aim to improve DIYAPS users’ ability to donate data for retrospective analysis for outcomes and future DIYAPS developments. This is designed for those who are interested in contributing to the DIYAPS community by donating their anonymized datasets, as described in WP1 [37].

The current workflow enables individuals using DIYAPS to upload data and contribute data anonymously to research, via the Open Humans platform. Users can either do a manual upload of their DIYAPS data, or they can utilize the “Nightscout Data Transfer” tool to pull data directly from Nightscout into Open Humans. Nightscout is an open-source remote monitoring platform commonly used for real-time visualization of disparate diabetes device data streams, also used for retrospective data analysis and to report generation, which is widely used by DIYAPS users [23]. Nightscout can capture behavioral data, such as exercise entries, temporary targets to adjust DIYAPS behavior, or meal entries, in addition to logging DIYAPS predictions and output at 5-min intervals, and it becomes a rich source for retrospective data analysis for research when donated to the OpenAPS Data Commons. However, not all individuals choose to use Nightscout, and other methods are therefore planned to increase the ease of data donation for research. The OPEN team also plans to add direct upload capabilities to one of the commonly used DIYAPS (AndroidAPS) that will authenticate directly with Open Humans and enable an additional data donation method to the OpenAPS Data Commons. The current uploading methods require the user to initiate any subsequent data uploads; both methods described above will permit users to opt in to enable automatic, regular data uploads. This both makes data donation easier and captures data that are often deleted, either accidentally or to free up storage space for the user. After enabling increased data donation with a wider and diverse population of DIYAPS users, we expect the OpenAPS Data Commons available dataset to be increased from about 115 users to an estimated 300 or more users. As the dataset is based on real-world data, there may be concerns about data integrity. However, data are processed and connected in many ways with complex decision trees, which makes it hard to falsify data. Furthermore, studies have shown that real-world data are as robust as, if not more robust than, data gathered in clinical trials with predefined selected populations [38].

Although current studies have shown that DIYAPS users achieve positive outcomes (clinical and quality of life) [26-31], there are areas for improvement and further iteration in terms of usability, algorithm features, and optimizing individual settings and preferences, as well as areas of statistical learning, which are applicable to all APS (DIY or commercially developed). With several hundred (see above) pseudonymized datasets, we expect to be able to break down outcome data into subcohorts to better quantify the impact of different algorithm choices and settings. This may include comparing outcomes among individuals using varying versions of algorithms over time, comparing different feature choices between and across individuals, and evaluating settings (such as specifications of medical devices and insulin, target values, specific feature use, and mealtime dosing behaviors). This will enable identification of less optimal use patterns in the real world, which will yield recommendations for prioritization of future improvement areas in development of DIYAPS, insight into the biggest needs of varying subcohorts, and this also indicates the most popular or most effective features in DIYAPS, which could also be translated and adapted and inform the use of commercially developed APS.

This WP is led by DL, PwD and founder of OpenAPS, and AT, PwD and developer of AndroidAPS.

### Work Package 4: Barriers to Scale-Up

This WP aims to explore the potential economic, social, cultural, legal, and political barriers to the scale-up of DIYAPS technology.

The first objective of this WP will be to examine the potential barriers to uptake experienced by PwD who are interested in DIYAPS but have so far opted not to build their own system, have opted out after an unsuccessful attempt, or otherwise chosen to discontinue DIYAPS. We observe that many people stay connected to the online DIYAPS community regardless of their initial choice to use DIYAPS or not, as indicated by the 10,000+ people participating in the main “Looped” group on Facebook, as compared with the estimated, approximately 3000 likely active users of DIYAPS. Members of the Facebook peer support group “Looped” and other related Facebook groups who have yet to build their own system will therefore be targeted through questionnaires that aim to capture the reasons for not doing so. The questionnaires will probe for indicators, such as lack of knowledge of the benefits of using DIYAPS, lack of confidence in one’s own information technology (IT) skills, affordability of technologies, time and effort needed to build DIYAPS, and other reasons for stopping DIYAPS, such as the availability of a commercial solution or lack of support from health care providers. The content of these questionnaires will be generated on the basis of a list of potential barriers to uptake, identified through the findings from WP2 [35,36] and qualitative in-depth interviews with a targeted sample of non-DIYAPS users. The overall outcome will be a greater understanding of the reasons why these individuals are not yet using DIYAPS or have chosen not to use DIYAPS, which will consequently help researchers to develop better ways of addressing barriers to access and adoption of APS.

The next defining point of this WP is committed to understanding the health equity implications associated with the progression of DIY technology-enabled solutions for chronic disease management. More specifically, we aim to capture the requirements of DIYAPS users with lower levels of IT literacy. This will be established through a series of one-to-one and focus group interviews. The interviews will inform the development of low-fidelity wireframes that can be demonstrated at user-experience design workshops for further feedback from less tech-savvy DIYAPS users. The result will be a series of use cases that will be made available for the benefit of DIYAPS developers and the medical device industry.

An additional aspect of WP4 is to examine the current extent to which there are observable social inequalities in terms of access to the technologies needed to build DIYAPS and how these inequalities might be addressed and minimized. From this perspective, we will utilize data from the T1International Out-of-Pocket Expenses survey to examine the out-of-pocket-expenses and other accessibility issues associated with DIYAPS-related technologies [39].

This WP is led by SO, PwD and sociologist at the School of Sociology, University College Dublin.

An overview of 4 scientific work packages is provided in Multimedia Appendix 1.

### Work Package 5: Communication and Dissemination

This WP will coordinate the training, communication, and dissemination activities of the OPEN project and its aims to ensure high visibility and direct impact in the community, involving relevant stakeholders. The objectives include (1) implementing dissemination actions and communication activities to targeted audiences, (2) promoting the project concept and vision by highlighting its unique structure connecting patient researchers and innovators with established research organizations through social media, workshops, our newsletter, and the project website, (3) ensuring technical and scientific dissemination of the project results through publications in journals and conferences, and (4) raising public awareness of the project’s objectives.

Different target audiences will be engaged with information adjusted carefully to their needs, raising awareness among those who can benefit from the project results and encouraging multistakeholder dialogue. The consortium has identified potential target groups, including the scientific community, clinicians and health care professionals, PwD, the DIYAPS community, policy makers and regulators, the medical and IT industry, and the general public.

All academic outputs that result from this project will be published in open-access journals and on the project’s public dissemination channels. Conference presentations at academic, clinical, and industry events provide an important opportunity for researchers to disseminate findings to multiple audiences. DIYAPS is currently a hot topic within diabetes care, and face-to-face interaction with conference delegates presents an opportunity to generate dialogue among both the proponents and critics of DIYAPS. This consortium will not only contribute to a body of evidence that will help to move the terms of the discussion forward but it will also ensure that the patient voice remains center stage.

Social media will also be used to ensure that the project findings reach multiple stakeholders and that the findings will influence decisions in public policy and professional practice. Twitter, LinkedIn, Facebook, and YouTube will be used as dissemination vehicles, providing a headline or snapshot summaries of key takeaway messages from the project outcomes, which will attract the attention of key stakeholders. Scheduled dissemination and communication activities will be held throughout the duration of the project to ensure constant information flow. Targeted messages will be distributed to the public to ensure that impact is maximized.

Finally, it is important to note that any technical innovations that might emerge or evolve from this project will be shared with the public, including DIYAPS developers, and it will ultimately be their decision whether to use the findings of OPEN as the basis to implement any changes in DIYAPS, as our role is focused solely on research and establishing an evidence base. Thus, the organizations’ efforts in this project are not directly involved in, and will remain separated from, any direct DIYAPS development.

This WP is led by BH, PwD and CEO of Dedoc Labs.

## Results

This project has received funding from the European Commission’s Horizon 2020 Research and Innovation Program, under the Marie Skłodowska-Curie Action Research and Innovation Staff Exchange grant agreement number 823902.

Initial results on clinical outcomes and patient-reported outcomes have been presented at the Advanced Diabetes Technologies and Treatments Conference in February 2019 in Berlin, Germany, and the American Diabetes Association 79th Scientific Sessions in San Francisco, the United States, in June 2019 [35,36,40,41]. A study on self-reported clinical outcomes of the pediatric population using DIYAPS has been recently published in *JMIR mHealth and uHealth* [26], showing improved glycemic outcomes across all pediatric age groups, which is in line with clinical trial results from commercially developed closed-loop systems.

## Discussion

DIYAPS represents an important case study in how increasingly informed and connected patients are shaping the direction of technological innovation in diabetes care and, potentially, for other areas of health care. As outlined above, researching this global movement poses unique challenges and opportunities, which necessitate a move away from traditional, top-down modes of scientific inquiry toward a more cooperative and interactive approach, which is largely driven by PwD themselves.

The OPEN project is uniquely placed to address these challenges, as it is a patient- and user-led research project that brings together an international, interdisciplinary, and intersectoral research group comprising health care professionals, technical developers, and biomedical and social scientists, many of whom live with diabetes and are active DIYAPS users. Thus, the OPEN consortium is poised to bring the benefits of expertise of all kinds combined with the prioritization and knowledge of on-the-ground patient needs in a way that, to the best of our knowledge, is not being addressed by other research projects and collaborative initiatives.

It is acknowledged that the nature of the OPEN project can pose potential limitations and challenges. For example, the clinical evaluation (WP1) may be potentially seen as lacking rigor because of its inclusion and use of user-provided data. However, it is precisely this aspect of our methodological approach which has the potential to make a significant contribution to the extant literature surrounding the effectiveness of APS technology. Most studies on APS technology, to date, have been carried out as randomized clinical trials in well-controlled clinical research settings. Therefore, little is known about efficacy of APS in real-world settings where outcomes are likely to be contingent on context [42]. Moreover, previous studies have, for the most part, focused on measuring biomedical disease specific–outcomes; therefore, many other consequences of using closed-loop systems, such as psychosocial outcomes, remain relatively poorly understood [43]. By being one of the first studies to generate evidence on the basis of reliable, user-provided data from all 3 DIYAPS (OpenAPS, AndroidAPS, and Loop) in real-world settings, this study will be an important complement to the existing clinically led research studies in the field.

In this regard, it is important to point out that both positive and negative forms of evidence surrounding DIYAPS will be valued equally by the OPEN team. Negative results provide important learning opportunities for the further development and diffusion of APS technology and for understanding what works, for whom, and under what set of circumstances [44]. This is why WPs around barriers (WP4), outcomes across a broad population (WP1 and WP2), and improvements to technology (WP3) have been designed. For example, qualitative studies exploring the lived experience of DIYAPS users (WP2) will seek out instances where individuals have used a closed-loop system but subsequently discontinued use. Finally, although negative results tend to be underreported in much of the traditional evaluation research literature [45], the OPEN team will openly share and attempt to publish all results from each WP.

Overall, by providing empirical evidence on DIYAPS, this project addresses the core user needs of the DIYAPS community. In addition, it will offer insights around accelerating improvements and diffusion of APS technology across the wider population of PwD. Disseminating project results in academic and nonacademic settings will help lower barriers among key stakeholders and encourage other researchers, policy makers, regulators, and the medical device industry to work together and innovate in a truly patient- and user-centric manner. We believe this new model of cooperation has the potential to have a profound impact on those living with diabetes, their families, health care systems, and society as a whole.

## Appendix

Multimedia Appendix 1 Overview of the four scientific work packages of the OPEN project.

## Abbreviations

APS: Artificial Pancreas System
CGM: continuous glucose monitoring
DIY: do-it-yourself
DIYAPS: Do-It-Yourself Artificial Pancreas Systems
HbA_1c_: glycated hemoglobin
IT: information technology
OPEN: Outcomes of Patients’ Evidence With Novel, Do-It-Yourself Artificial Pancreas Technology
OpenAPS: Open-Source Artificial Pancreas System
PwD: people with diabetes
T1D: type 1 diabetes
WP: work package
