# Supplementary figures and images for: Evidence on User-Led Innovation in Diabetes Technology (The OPEN Project): Protocol for a Mixed Methods Study
Source: JMIR Res Protoc. 2019 Nov 19;8(11):e15368. doi: 10.2196/15368 (PMC6891827; doi:10.2196/15368)

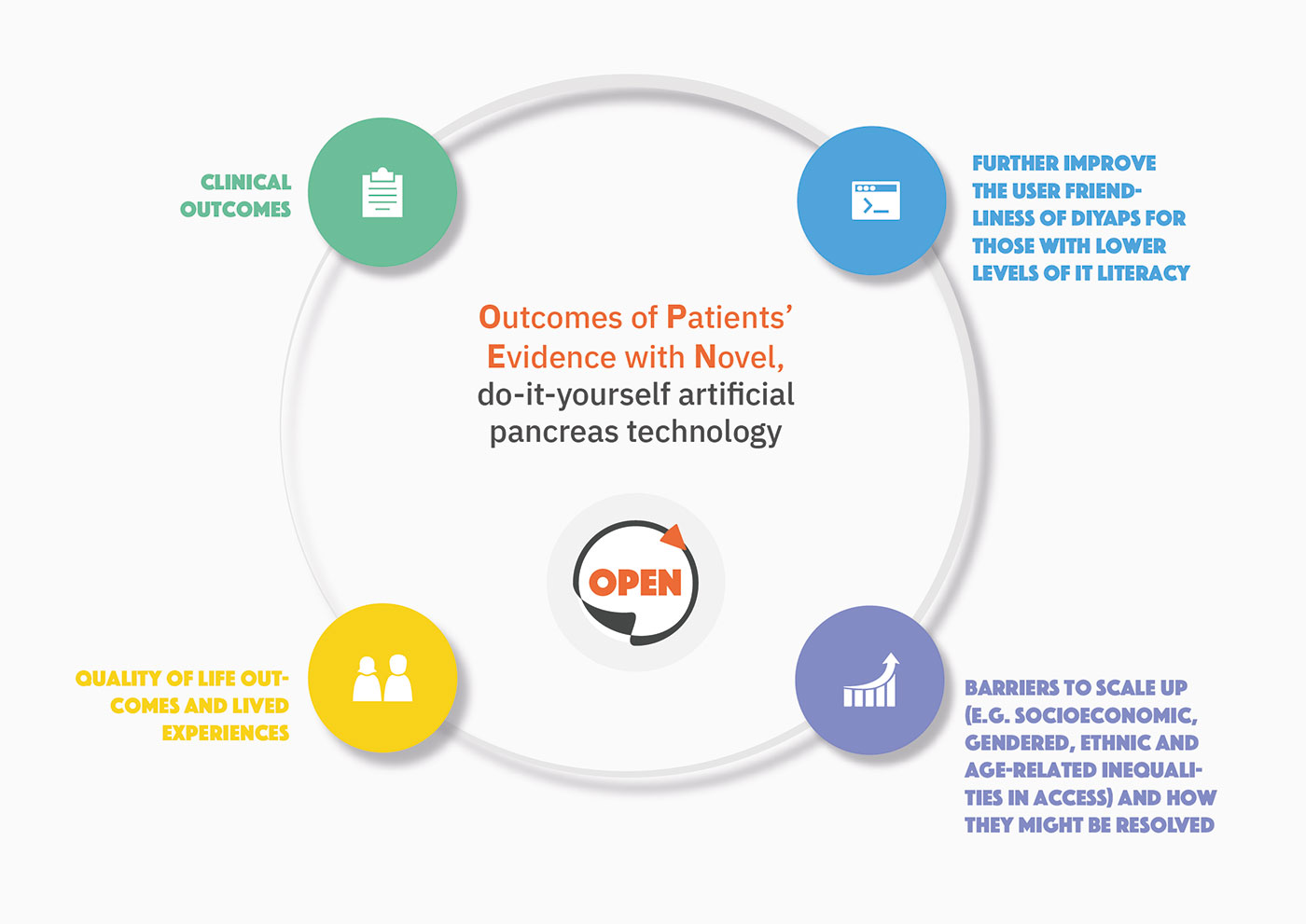

Supplement: Multimedia Appendix 1 [file resprot_v8i11e15368_app1.png]
